# Promoted room temperature NH_3_ gas sensitivity using interstitial Na dopant and structure distortion in Fe_0.2_Ni_0.8_WO_4_

**DOI:** 10.3389/fchem.2024.1480294

**Published:** 2024-10-23

**Authors:** Jong Hyun Lee, Seung Yong Lee, Myung Sik Choi, Kyu Hyoung Lee

**Affiliations:** ^1^ Department of Materials Science and Engineering, Yonsei University, Seoul, Republic of Korea; ^2^ Department of Nano and Advanced Materials Science and Engineering, Kyungpook National University, Sangju, Republic of Korea; ^3^ Yonsei-KIST Convergence Research Institute, Seoul, Republic of Korea

**Keywords:** metal oxide, NiWO_4_, gas sensor, NH_3_ gas, co-doping

## Abstract

The demand for gas-sensing operations with lower electrical power and guaranteed sensitivity has increased over the decades due to worsening indoor air pollution. In this report, we develop room-temperature operational NH_3_ gas-sensing materials, which are activated through electron doping and crystal structure distortion effect in Fe_0.2_Ni_0.8_WO_4_. The base material, synthesized through solid-state synthesis, involves Fe cations substitutionally located at the Ni sites of the NiWO_4_ crystal structure and shows no gas-sensing response at room temperature. However, doping Na into the interstitial sites of Fe_0.2_Ni_0.8_WO_4_ activates gas adsorption on the surface via electron donation to the cations. Additionally, the hydrothermal method used to achieve a more than 70-fold increase in the surface area of structure-distorted Na-doped Fe_0.2_Ni_0.8_WO_4_ powder significantly enhances gas sensitivity, resulting in a 4-times increase in NH_3_ gas response (R_g_/R_a_). Photoluminescence and XPS results indicate negligible oxygen vacancies, demonstrating that cation contributions are crucial for gas-sensing activities in Na-doped Fe_0.2_Ni_0.8_WO_4_. This suggests the potential for modulating gas sensitivity through carrier concentration and crystal structure distortion. These findings can be applied to the development of room-temperature operational gas-sensing materials based on the cations.

## 1 Introduction

The hazardous nature of certain gases, which are poisonous, flammable, and volatile, has garnered serious attention in both industrial areas and indoor environments. Ammonia gas (NH_3_) is a representative reducing gas commonly used in the fertilizer and food industries, while NO_x_ gases are byproducts of fossil fuel combustion. Despite its useful applications, NH_3_ poses significant health risks, contributes to water pollution, and carries a risk of explosion at high concentrations in confined spaces. This has led to the establishment of permissible exposure limits for NH_3_, which are set at up to 50 ppm for durations of less than 8 h (Li et al., 2018; Kim et al., 2002; Wang et al., 2017; Choudhari and Jagtap, 2023; Natarajamani et al., 2024; Duy et al., 2023; Wang et al., 2018). To monitor NH_3_ leakage and concentration, numerous chemiresistive gas sensing materials using metal oxides (MO) have been developed. These materials are favored for their low cost and rapid response times, and they exhibit significant changes in electrical resistance when exposed to varying concentrations of NH_3_ gas (Wang et al., 2017; Choudhari and Jagtap, 2023; Natarajamani et al., 2024; Duy et al., 2023; Natarajamani et al., 2024; Late et al., 2014; Shi et al., 2024; Wagh et al., 2006).

Metal oxides (MO) exhibit large band gaps due to the strong ionic bonding between cations and oxygen anions, which results in semiconductor behavior through changes in surface carrier concentration when NH_3_ gases are adsorbed on the MO surface (Choudhari and Jagtap, 2023; Natarajamani et al., 2024; Duy et al., 2023; Natarajamani et al., 2024; Hwang et al., 2023). Most advancements in MO-based gas-sensing materials have focused on modulating oxygen vacancy concentration and morphological approaches (Choi et al., 2021; Jin et al., 2022; Lee et al., 2023; Kim et al., 2023; Yamazoe, 2005; Jia et al., 2014; Simon et al., 2001; Zakrzewska, 2001; Franco et al., 2022; Eranna et al., 2024; Ji et al., 2019; Chen et al., 2013; Dey, 2018). Numerous reports have shown significant improvements in gas sensitivity, which depends on the presence of oxygen anions at gas adsorption sites. This is often achieved by enlarging the surface area and increasing oxygen vacancy concentration, which gives an advantage in using these systems as cost-effective and suitable for manufacturable integrated gas sensing systems (Tang et al., 2022; Srinivasan et al., 2019; Shah et al., 2022). However, controlling the appropriate oxygen vacancy concentration is challenging due to the random and non-uniform generation of defects or morphological variations in micro- and nano-sized MO particles, leading to lower reproducibility in gas sensing performance. Additionally, the strong insulating nature of MO limits the operational temperature for gas sensing to above several hundred degrees Celsius. This is due not only to the presence of H_2_O and -OH groups on the MO surface but also to the need for energy that supports activated carrier transfer through the large band gap. While heating the device to the required operational temperature results in higher electrical power consumption, there is a growing demand for room-temperature operational gas sensing devices, which is driving the development of advanced materials (Wang et al., 2005; Chen et al., 2019; Naikoo et al., 2017; Nath et al., 2024).

In this work, we suggest the room temperature NH_3_ gas sensing materials by using cation charge state and surface effect change in Fe_0.2_Ni_0.8_WO_4_ crystal structure, which the substitutional Fe doping in the strongly correlated electron system of NiWO_4_ (Han et al., 2024; Suh et al., 2024), demonstrate the higher cation contributed gas adsorption site. From the no gas sensing response from Fe_0.2_Ni_0.8_WO_4_ crystal, the electron-doped from interstitial Na doping in free space exhibits originated from the changed cation charge state and measured gas sensing response. In addition, the higher surface area synthesized by the hydrothermal method exhibits the 4-fold improved NH_3_ gas response and fast response/recovery times via changed cation-oxygen anion vibration mode without oxygen vacancy difference. These results introduce the possibility of dominant cations contributing to room-temperature operational gas sensing performance in MO particles.

## 2 Materials and methods

### 2.1 Material synthesis

The Fe_0.2_Ni_0.8_WO_4_ powders were synthesized using a solid-state reaction. High purity Fe_2_O_3_ (Kojundo Chemical Lab, 99.9%), NiO (Kojundo Chemical Lab, 99.97%), and WO_3_ (Kojundo Chemical Lab, 99.9%) powders were mixed in a 0.1: 0.8: 1 mole ratio in an alumina mortar. The mixed powders performed a heat treatment in an electric box furnace at 1,050°C for 12 h. To synthesize the 0.05% of Na doped Fe_0.2_Ni_0.8_WO_4_, we add 0.025% mole ratio of the Na_2_CO_3_ powder (Kojundo Chemical Lab, 99%), as following the equation 0.025: 0.1: 0.8: 1 = Na_2_CO_3_: Fe_2_O_3_: NiO: WO_3_.

The hydrothermally synthesized Na-Fe_0.2_Ni_0.8_WO_4_ is used Na_2_WO_4_.2H_2_O, FeCl_2_, and NiCl_2_.6H_2_O precursor and totally dissolved in Di-water. After that, a Teflon container containing the substance was performed hydrothermal synthesis using an autoclave under 180°C for 6 h. The synthesized wet samples were carried out overnight in the dry process in the vacuum oven at 60°C. Sequentially, dried powders were calcinated at 600°C for 1 h.

### 2.2 Material characterization

Morphological measurements were conducted by scanning electron microscopy (SEM, JEOL-7800F, JEOL Ltd.). The crystal structure characterization was performed using X-ray diffraction (XRD, Smart Lab, Rigaku) with Cu Kα radiation. Chemical bonding states were analyzed via X-ray photoelectron spectroscopy (XPS, K-alpha, Thermo Fisher Scientific Co.) Raman spectroscopy (LabRam Aramis, Horiba Jovin Yvon) and FT-IR measurement (Invenio, Brucker) were utilized to confirm the vibration mode of NiWO_4_.

### 2.3 Evaluate the gas-sensing performance

A 2-probe electrode configuration was employed for the gas sensing analysis. Fe_0.2_Ni_0.8_WO_4_, Na- Fe_0.2_Ni_0.8_WO_4_, and Hydrothermal synthesized Na-Fe_0.2_Ni_0.8_WO_4_ properties put on the gold electrodes positioned on an alumina substrate with 0.2 mL of ethanol on synthesized powders and physically pressed the powder to fix the powder on the substrate. The gas-sensing performance of the fabricated sensors was evaluated within a custom-built chamber equipped with mass flow controllers, maintaining a fixed flow rate of 500 standard cubic centimeters per minute using air as the carrier gas. The sensors were exposed to target gas concentrations ranging up to 20 ppm for 100 s, followed by a recovery period in the air for 200 s at 30°C. The resistance values in air (R_a_) and upon exposure to the target gases (R_g_) were recorded, and the sensor response (R_g_/R_a_) was determined by calculating the ratio of resistance values in ambient air to under target gas exposure conditions. Gas sensing measurements were performed for various gases, including NH_3_, H_2_S, NO_2_, SO_2_, Benzene, p-Xylene, HCHO, and Acetone.

## 3 Results

### 3.1 Crystal structure analysis

**FIGURE 1:**
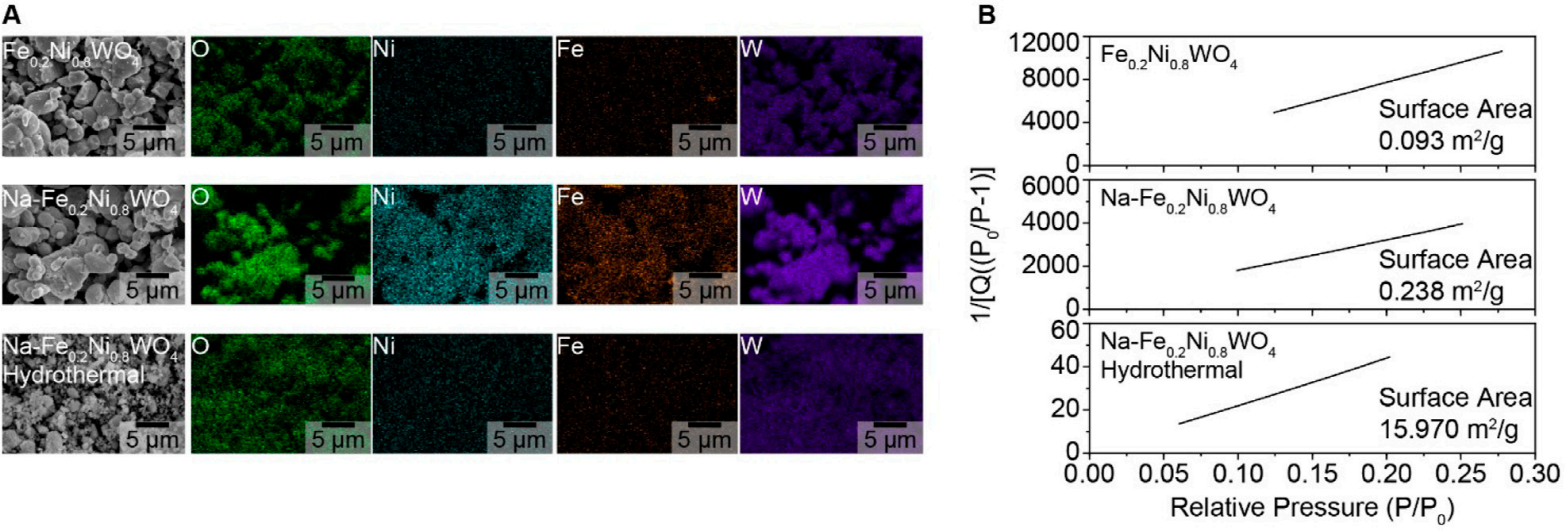
Morphological properties of synthesized powders for Fe_0.2_Ni_0.8_WO_4_, Na-Fe_0.2_Ni_0.8_WO_4_, and hydrothermal synthesized Na-Fe_0.2_Ni_0.8_WO_4_ (Na-Fe_0.2_-Hydro). **(A)** SEM/EDS results. **(B)** Krypton-BET results.

The morphological shape of Fe_0.2_Ni_0.8_WO_4_, Na- Fe_0.2_Ni_0.8_WO_4_ (Na-Fe_0.2_), and Na-Fe_0.2_Ni_0.8_WO_4_ hydrothermal (Na-Fe_0.2_-Hydro) synthesized powders is shown in SEM and EDS results in Figure 1A. EDS results show the evenly mixed elements such as O, Ni, Fe, and W. The surface area measured by Krypton Brunauer-Emmett-Teller (BET) analysis exhibits the 0.093 m^2^/g of Fe_0.2_Ni_0.8_WO_4_ and 0.238 m^2^/g of Na- Fe_0.2_Ni_0.8_WO_4_ through the solid-state synthesized method. However, the hydrothermally synthesized Na-Fe_0.2_Ni_0.8_WO_4_ powder shows a surface area more than 67 times larger (15.970 m^2^/g), which is related to the smaller particle size of Na-Fe_0.2_-Hydro sample. The higher surface area of Na-Fe_0.2_-Hydro samples is fabricated by the hydrothermal synthesis method, which makes it possible to precise control over compositions through liquid or multiphase reactions. Hydrothermal synthesis is a widely used solution-based method for preparing nanomaterials across a broad temperature range, allowing control over material morphology, including the synthesis of nanoparticles, nanorods, nanotubes, hollow nanospheres, etc., (Darr et al., 2017) A detailed analysis of the cationic ratio using ICP analysis for all samples, as shown in Table 1, indicates an approximately 0.2 Fe and 0.8 Ni ratio with W deficiency. The Na concentration shows 0.014 at% in Na-Fe_0.2_-Hydro and 0.059 at% in Na-Fe_0.2_ samples, respectively. Considering the higher surface area manifested by using hydrothermally synthesized powder, it commonly shows higher microscopic and induces crystal structure distortion from intrinsic composition, which drives the higher gas absorption site for higher gas sensitivity.

**TABLE 1:** ICP results of each cation at% in synthesized powders.

	Na (at%)	Fe (at%)	Ni (at%)	W (at%)
Na-Fe_0.2_-Hydro	0.014	12.175	53.954	33.857
Na-Fe_0.2_	0.059	11.497	51.588	36.856
Fe_0.2_Ni_0.8_WO_4_	-	10.945	54.003	35.052

**FIGURE 2:**
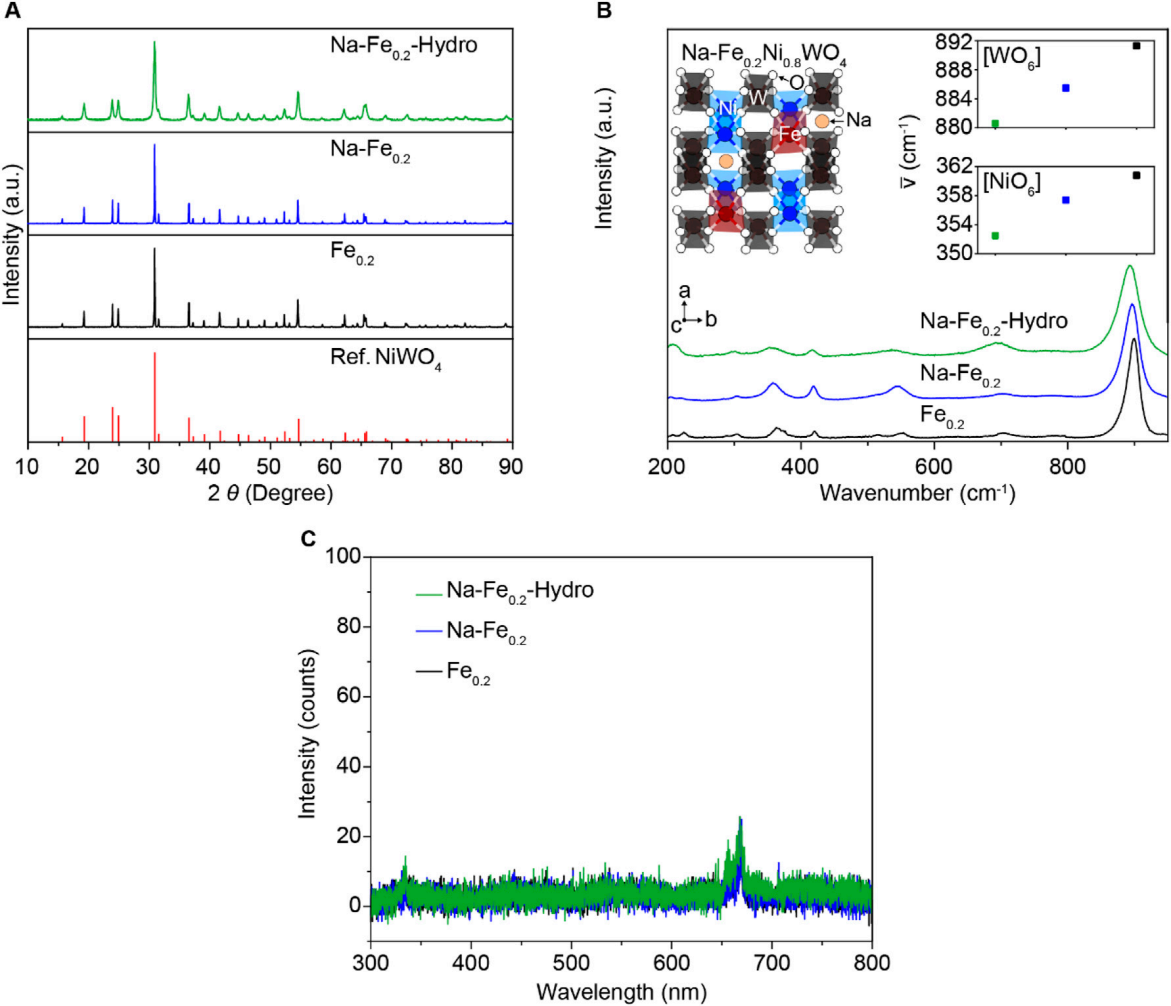
Crystal structure analysis of Na-Fe_0.2_-Hydro, Na-Fe_0.2_, and Fe_0.2_Ni_0.8_WO_4_ powders. **(A)** XRD results. **(B)** Raman results. Inset is a schematic illustrated Na-Fe_0.2_Ni_0.8_WO_4_ crystal structure. **(C)** Photoluminescence results.

To understand the detail of structure change depending on the N a doping and the nano-sized structure manipulation process, we performed crystal structure analysis for all samples to identify the effect of the Na dopant and increased surface area such as XRD, Raman, and PL spectroscopy. Figure 2A exhibits the powder XRD pattern, compared with the reported NiWO_4_ XRD results. All samples exhibit the single phase of XRD patterns without impurities as the NiWO_4_ reference (Han et al., 2024; Suh et al., 2024). Thus, the doping elements, such as Fe and Na, are well-dissolved in the NiWO_4_ crystal structure. The Raman spectroscopy results in Figure 2B demonstrate the different bonding vibration modes between cation-oxygen anions. The detailed view on the left side of the inset figure describes the crystal structure of Na-Fe_0.2_Ni_0.8_WO_4_. The P2/c space group of the monoclinic wolframite structure NiWO_4_ is composed of corner-shared [NiO_6_] and [WO_6_] octahedral structures (Han et al., 2024; Suh et al., 2024), which have substitutional dopant Fe at the Ni site, and the Na are located in the interstitial free space between each of octahedral structures. Among the diverse vibration modes, two Raman active modes were observed: the highest intensity peaks at 880–892 cm^−1^ ranged correspond to the symmetric vibrations of W–O bonding for symmetric vibrations, whereas the Raman peaks near 350–362 cm^−1^ are the symmetric vibrations of [NiO_6_] or [FeO_6_] octahedral structures. The red-shift when interstitial Na doping on Fe_0.2_Ni_0.8_WO_4_ indicates the lower bonding strength between cations and O via partially located Na in the free space of crystal structure sequentially the fabricated larger surface area also causes a red peak shift compared to the Fe_0.2_Ni_0.8_WO_4_ samples that carried the local lattice structure distortion such as lower crystallinity [NiO_6_] and [WO_6_] octahedral structures from the higher surface area than intrinsic Fe_0.2_Ni_0.8_WO_4_ powders. Although hydrothermally synthesized MO powders have tended to fabricate the higher crystallographic defects from significantly higher surface area and generate external carriers (called F-center), the Fe_0.2_Ni_0.8_WO_4_, (Na-Fe_0.2_), and (Na-Fe_0.2_-Hydro) powders exhibit the negligible intensity in the 300–800 nm wavelength range for all samples (as shown in Figure 2C), demonstrating the ignorable oxygen vacancy in the crystal structure, which is matched with the XPS results.

**FIGURE 3:**
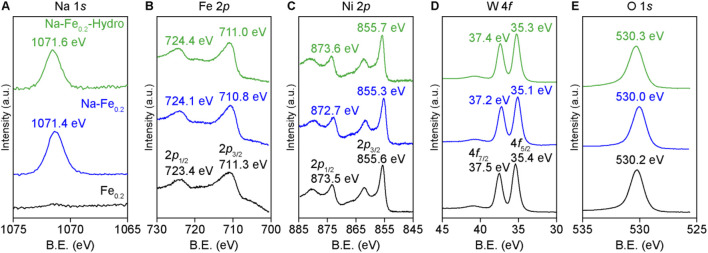
XPS results of each cation in synthesized powders. **(A)** Na 1*s*. **(B)** Fe 2*p*. **(C)** Ni 2*p*. **(D)** W 4*f*. **(E)** O 1*s*.

The XPS results in Figures 3A–E show the chemical states of each element in the Na-Fe_0.2_-Hydro, Na-Fe_0.2_, and Fe_0.2_Ni_0.8_WO_4_ powders. The Na 1s binding energies are 1,071.6 eV for Na-Fe_0.2_-Hydro and 1,071.4 eV for Na-Fe_0.2_ samples, similar to unionized Na states and lower than the binding energy of Na_2_O. Regarding the binding energies of the cations, specifically Fe 2p, Ni 2p, and W 4f, are shown lower binding energies at the maximum intensity of each peak when Na is doped at the interstitial site compared to the intrinsic Fe_0.2_Ni_0.8_WO_4_ and Na-Fe_0.2_Ni_0.8_WO_4_ structure due to the electron doping to Fe_0.2_Ni_0.8_WO_4_ structure via the Na dopant acting as electron donor.

However, the similar binding energies of Na-Fe_0.2_-Hydro and Fe_0.2_Ni_0.8_WO_4_ samples or blue shift than the Na-Fe_0.2_ powders indicate an offset between the chemical states due to electron donation and surface effects that can tune the cations charge state change, which presents the higher binding energy shifting on cations charge state owing to the larger surface area (Tsunekawa et al., 2000). Additionally, the O 1s results show a symmetrical peak shape near 530 eV, which corresponds to a negligible oxygen vacancy effect in all samples, as measured by the PL results. Thus, the difference in gas sensing performance among Na-Fe_0.2_-Hydro, Na-Fe_0.2_, and Fe_0.2_Ni_0.8_WO_4_ powders predominantly depends on the charge states of the cations and the crystal structure distortion, which are induced by the doping and the formation of a larger surface area.

### 3.2 Room temperature gas sensing results

**FIGURE 4:**
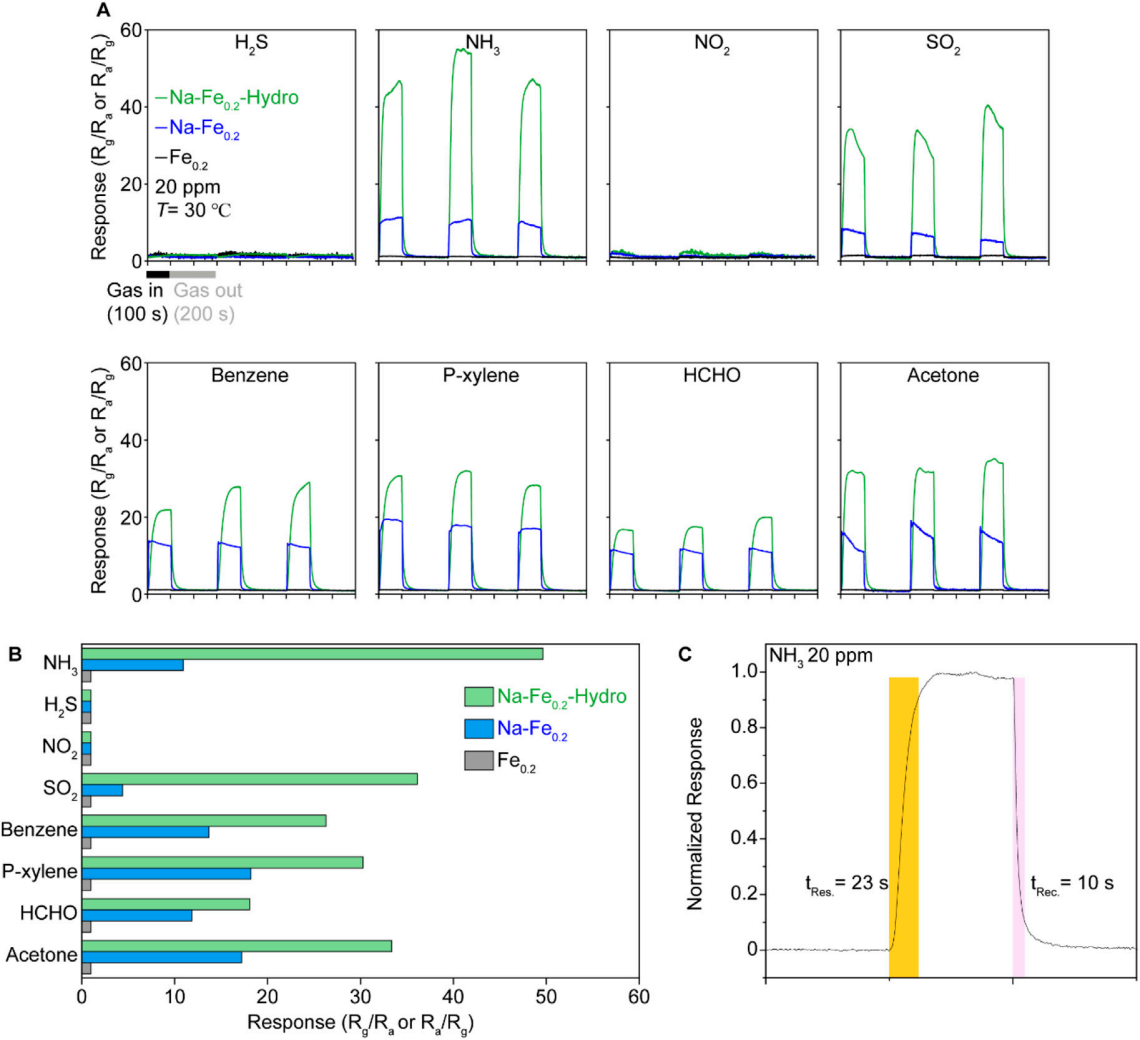
The room temperature gas sensing results of Na-Fe_0.2_-Hydro, Na-Fe_0.2_, and Fe_0.2_Ni_0.8_WO_4_ samples under 20 ppm of diluted gases. **(A)** The response is derived by electrical resistance difference under air and each of gases such as NH_3_, H_2_S, NO_2_, SO_2_, Benzene, P-xylene, HCHO, and Acetone. **(B)** Plot of gas selectivity via average response-analyte gases. **(C)** Detailed view of gas response time and recover times on Na-Fe_0.2_-Hydro samples under 20 ppm of NH_3_.

The gas sensing performance is illustrated in Figures 4A, B. The gas sensing measurements for analyte gases at 20 ppm were conducted at room temperature using Au electrode coated Al_2_O_3_ substrate and sample powders are fixed by physically pressed on the substate with few drops of ethanol. The Fe_0.2_ sample presented almost none of the gas response under all gases such as NH_3_, H_2_S, NO_2_, SO_2_, Benzene, p-Xylene, HCHO, and Acetone, which presented a 10–20 range of gas sensing response and vague gas selectivity. However, both Na-Fe_0.2_-Hydro and Na-Fe_0.2_ samples exhibit a reasonable gas sensing response except under NO_2_ and H_2_S, which maintain negligible gas sensing response. The emergence of gas sensing functionality on the Na-Fe_0.2_ indicates that the incorporation of Na plays a crucial role in activating the gas adsorption site by carrier doping, which follows the interstitially located alkali metal acting as a carrier donor to matrix materials. On the other hand, the Na-Fe_0.2_-Hydro sample demonstrates a remarkable response (R_g_/R_a_) at room temperature. Among the improved gas sensing response in the Na-Fe_0.2_-hydro sample, over 40 gas sensing responses under NH_3_, which is more than four times higher than the NH_3_ gas sensing response (∼10 R_g_/R_a_) observed in the Na-Fe_0.2_ sample. This substantial improvement in the gas response can be attributed to the synergistic effects of carrier doping and the distorted crystal structure through the hydrothermal synthesis method. The response and recovery times, shown in Figure 2C, indicate that the sensor quickly reaches 90% of its maximum response within 23 s after the start of gas exposure. This rapid response time is essential for real-time monitoring and quick detection of gas leaks. Additionally, the recovery time is 10 s, returning to 10% of its response after the gas exposure is turned off. This quick recovery is crucial for ensuring that the sensor can be reused for continuous monitoring without long delays between measurements.

These results highlight the effectiveness of Na doping and the hydrothermal synthesis method in significantly enhancing the gas sensing performance of Fe_0.2_Ni_0.8_WO_4_. Furthermore, considering the higher gas physisorption behavior on MO shown at room temperature due to the lower activation energy of the chemical reaction between the gas and the MO surface, the weak oxygen vacancy effect on both the solid-state synthesized and hydrothermally synthesized samples suggests that the gas adsorption site is contributed by the cations. This is generated by the modified crystal structure and cation charge states, which are key factors contributing to the improved sensitivity and selectivity toward NH_3_ gas sensing. The rapid response and recovery times further demonstrate the potential of these materials for practical applications in real-time gas sensing at room temperature for conventional MO composition.

## 4 Conclusion

The gas sensing results of Fe_0.2_Ni_0.8_WO_4_, tuned by electron doping from interstitial Na and the distortion of the crystal structure, show not only the emergence of gas sensing functionality but also improved sensitivity and NH_3_ selectivity at room temperature. Additionally, faster response times (23 s) and recovery times (10 s) under a diluted NH_3_ gas environment demonstrate the applicability of these gas sensing materials, which utilize a few micro-sized MO powders operated by cations. This implies the key role of cations in providing gas absorption sites and facilitating carrier transfer between the target gas and the material surface. This gas-sensing mechanism could inspire the development of various gas-sensing materials that are less affected by oxygen vacancies.

## Data Availability

The raw data in this article will be available on request.
